# Evaluation and Comparison of Multiple Aligners for Next-Generation Sequencing Data Analysis

**DOI:** 10.1155/2014/309650

**Published:** 2014-03-23

**Authors:** Jing Shang, Fei Zhu, Wanwipa Vongsangnak, Yifei Tang, Wenyu Zhang, Bairong Shen

**Affiliations:** ^1^Center for Systems Biology, Soochow University, 1st Shizi Street, Suzhou, Jiangsu 215006, China; ^2^Suzhou Institute of Nano-Tech and Nano-Bionics, Chinese Academy of Sciences, Suzhou 215123, China; ^3^School of Computer Science and Technology, Soochow University, Suzhou 215006, China

## Abstract

Next-generation sequencing (NGS) technology has rapidly advanced and generated the massive data volumes. To align and map the NGS data, biologists often randomly select a number of aligners without concerning their suitable feature, high performance, and high accuracy as well as sequence variations and polymorphisms existing on reference genome. This study aims to systematically evaluate and compare the capability of multiple aligners for NGS data analysis. To explore this capability, we firstly performed alignment algorithms comparison and classification. We further used long-read and short-read datasets from both real-life and *in silico* NGS data for comparative analysis and evaluation of these aligners focusing on three criteria, namely, application-specific alignment feature, computational performance, and alignment accuracy. Our study demonstrated the overall evaluation and comparison of multiple aligners for NGS data analysis. This serves as an important guiding resource for biologists to gain further insight into suitable selection of aligners for specific and broad applications.

## 1. Introduction

With a very high speed, large-scale sequencing reads, and drastically reduced costs available, next-generation sequencing (NGS) technology has appeared to be very fashionable [1]. There are a large number of studies that have successfully used NGS technology for their investigations under biological contexts of interests. For instance, in the nucleotide level, NGS technology is effectively used for genome evolution and genetic variation studies [2, 3]. In the transcription level, it is often applied for microRNA discovery and genomewide expression analysis [4, 5]. For the protein level, ChIP-sequencing technology is efficiently used for the identification of transcription factor binding sites [6] and histone modification patterns [7, 8]. Through a number of studies mentioned, undoubtedly, NGS represents a great powerful technology today which allows the massive number of sequencing reads to become available for only a short period and routinely be used for various genomewide association studies by aligning and mapping on the reference genome [9]. In recent years, there are several different aligners developed and further used for aligning and mapping for NGS data analysis. For examples, there are Mapping and Assembly with Qualities (MAQ) developed by Li et al. [10], Basic Oligonucleotide Alignment Software (BOAT) developed by Zhao et al. [11], Periodic Seed Mapping (PerM) developed by Chen et al. [12], Short Oligonucleotide Analysis Package (SOAPv2) developed by Li et al. [13, 14], and Global Alignment Short Sequence Search Software (GASSST) developed by Rizk and Lavenier [15].

In order to align and map NGS data using aligners, biologists often randomly select aligner without concerning to its feature, performance, and accuracy. Sequence variations and sequencing errors usually exist in the reference genome (e.g., repetitive regions and polymorphisms); hence, NGS reads frequently showed poor aligning and mapping [16]. In this case, if an unsuitable aligner is selected with existing repetitive regions and polymorphisms, the results may then convey error messages and mislead interpretation of biological outcome. It is therefore valuable for the biologists to consider the capability of individual software tool in terms of its feature, performance, and accuracy [5, 17]. This study is aimed to systematically evaluate and compare the capability of multiple aligners for NGS data analysis. Initially, we classified multiple aligners based on their developed algorithms. Here, hash table-based algorithm and Burrows-Wheeler Transform- (BWT-) based backtracking algorithm were considered. Under these two algorithms, we then selected favorable aligners for comparative analysis and further evaluation focused on three criteria (i.e., application-specific alignment feature, computational performance, and alignment accuracy). Literature searching and our own programming implementation were performed in order to evaluate different application-specific alignment features. Real-life datasets sampled from different organisms, including long-read datasets from Roche 454 sequencing platform and short-read datasets from Illumina sequencing platform, were used for comparative analysis of multiple aligners for computational performance evaluation. To further evaluate alignment accuracy, our generated* in silico *short-read and long-read datasets based on varying sequencing characteristics were used for comparison of multiple aligners. Through the end, the overall evaluation and comparison of multiple aligners with respect to the three criteria could guide the biologists for suitable selection of aligners for NGS data analysis for proper interpretation through different biological questions.

## 2. Results and Discussion

### 2.1. Algorithm-Based Classification of Multiple Aligners

**Figure 1:**
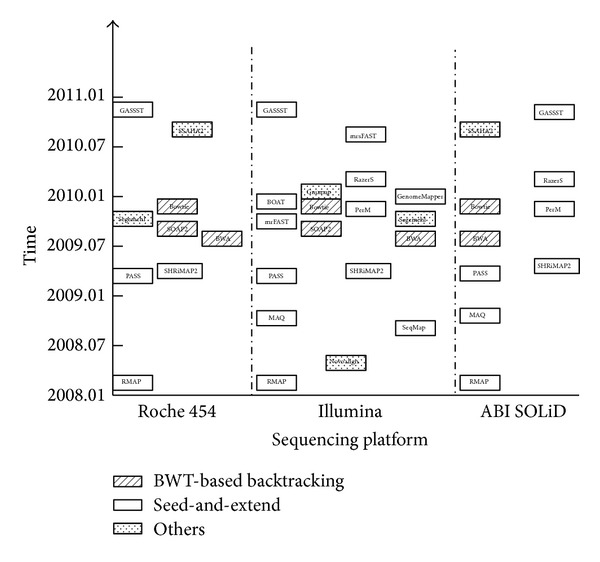
Aligners based on algorithms classification across different NGS platforms. Rectangles with different gray scales represent hash table-based algorithm, BWT-based backtracking algorithm, and other algorithms, individually. Aligners for specific types of data generated by different sequencing platforms are separately shown in three columns, namely, Roche 454, Illumina, and ABI SOLiD.

Currently, three NGS platforms, namely, Roche 454, Illumina, and ABI SOLiD, are employed at large extent, of biomedical researches. SOLiD platform generated two-base encoding data to discriminate between sequencing errors and SNPs [18], while Roche 454 platform has the ability to generate reads with length up to 500 nt or even longer, which is especially specific for de novo sequencing and resequencing [16]. Illumina platform is capable of producing hundreds of millions of much shorter reads at faster speed and lower cost than others. In addition, Roche 454 platform is more likely to have higher sequencing error rate of insertions and deletions, while Illumina platform typically possesses higher sequencing error rate of mismatches [19]. To adapt to high-throughput data from three NGS platforms, multiple aligners were designed with various algorithms. According to two main strategies employed behind the multiple algorithms, multiple aligners for NGS data were classified as the hash table-based algorithm and the BWT-based backtracking algorithm. As presented in Figure 1, we show 19 aligners based on these two algorithms for the three NGS platforms. According to the popularity of multiple aligners (see Supplementary File 3 in Supplementary Material available online at http://dx.doi.org/10.1155/2014/309650), the aligners, like RMAP, SeqMap, MAQ, SHRiMP2, BWA, SOAP2, and Bowtie, are popular for Illumina platform. RMAP, SHRiMP2, BWA, SOAP2, Bowtie, and SSAHA2 are widely applied for Roche 454, while RMAP, MAQ, SHRiMP2, BWA, Bowtie, and SSAHA2 are favorable for SOLiD platform.

To describe the hash table-based algorithm, initially, this algorithm accurately aligns massive data volumes produced by the present sequencing machines following an essential multistep strategy, called seed-and-extend [20]. To quickly identify limited subset of possible read mapping locations in the reference genome, the first step in the hash table-based algorithm is an attempt to localize the common k-mer substrings shared by both reads and genome sequences through the hash tables, called seeds detection. This step is specifically designed for accelerating high-throughput short reads. To determine the exact locations of the reads in the reference genome, the second step is subsequently to perform an extended alignment of seeds with slower and more accurate dynamic programming algorithm, such as Smith-Waterman [21] or Needleman-Wunsch algorithm. The aligners for NGS data analysis which were classified together in the hash table-based algorithm include SeqMap [22], PASS [23], MAQ [10], GASSST [15], RMAP [16], PerM [12], RazerS [24, 25], microread Fast Alignment Search Tool (mrFAST) [26], microread (substitutions only) Fast Alignment and Search Tool (mrsFAST) [27], GenomeMapper [28], and BOAT [11].

However, diverse strategies for seeds detection cause a distinction among multiple alignment algorithms. To handle the reads alignment with errors (e.g., mismatches and indels), RMAP, MAQ, SeqMap, and SOAP2 are based on the pigeonhole principle to chop the reads into small pieces to be perfectly matched to the reference genome for noncandidate filtration during seeds detection process [10, 16, 22, 29]. Meanwhile, SHRiMP2 [30, 31] and RazerS are implemented from another similar strategy, called q-gram filter. This is an extension of the pigeonhole principle to chop the reads into overlapping pieces to be matched for noncandidate filtration [24, 30].

Furthermore, the capability to align reads with many errors existing is also important bottleneck because pieces of reads are chopped so small with increased errors that lead to multiple match locations in the reference genome [32]. Thus, the algorithm based on the idea of spaced seeds, which is utilizing seeds with nonconsecutive matches in seed detection phase [12, 15, 30, 33, 34], has been used, for instance, in PerM, SHRiMP2, RazerS, BOAT, and GASSST.

In contrast, the BWT-based backtracking algorithm aligns the entire reads instead of the seeds of reads against the substrings sampled from the reference genome. To enable rapid read searching, this algorithm stores all the suffixes of reference genome sequence based on a certain representation of data structure, including prefix/suffix tree, suffix array, and Ferragina-Manziniuse algorithm-based index (FM index) [35]. This strategy is also used to solve alignment to multiple identical copies in the reference genome sequence efficiently, which is superior to the hash table-based algorithm. To reduce the memory occupation of the data structures as mentioned above, BWT [36–38], a reversible data compression algorithm, has been used to reorder the reference genome sequence for data structure compression. Thus, BWT-based backtracking algorithm retrieves the whole BWT-based suffix array for reads aligning and mapping with rapid searching and few memory requirements. Currently, SOAP2, BWA [39, 40], and Bowtie [37] were classified together in the BWT-based backtracking algorithm. For example, Bowtie employs BWT algorithm to compress FM index, while BWA constructs BWT-based suffix array for rapid subsequence search. In conclusions, the hash table-based algorithm and BWT-based backtracking algorithm showed contradiction of the alignment algorithms. To further compare individual aligner with these two alignment algorithms mentioned above, we performed evaluation and comparative analysis of these aligners in terms of computational performance, alignment accuracy, and application-specific features. The results are described as follows.

### 2.2. Application-Specific Features of the Multiple Aligners

Application-specific features were mined and collected through literature searching and our own programming implementation (see Section 4). Interestingly, we found that most of the aligners could support paired-end alignment for repetitive regions mapping excluding BOAT, GASSST, Gnumap [41], GenomeMapper, and SeqMap. With regard to gapped alignment, it was clearly shown that only 5 aligners lacked the function for SNPs and structural variation discovery, namely, Bowtie, mrsFAST, MAQ, RMAP, and SSAHA2 [42]. For bisulfite alignment used in ChIP-Seq data analysis, only Gnumap, mrsFAST, Novoalign (http://www.novocraft.com/), RMAP, and Segemehl [19] were demonstrated to support this function. To summarize, it was clear that Novoalign and Segemehl beneficially supported wide applications of multiple alignment features analysis, namely, gapped alignment, paired-end alignment, and bisulfite alignment.  Table 1 described different application-specific features among multiple aligners.

### 2.3. Computational Performance Evaluation Using Real-Life Datasets

To evaluate computational performance of individual aligner, we considered three factors that were computation time, maximum memory usage, and mapped read counts as follows.

#### 2.3.1. Computation Time Comparison

**Figure 2:**
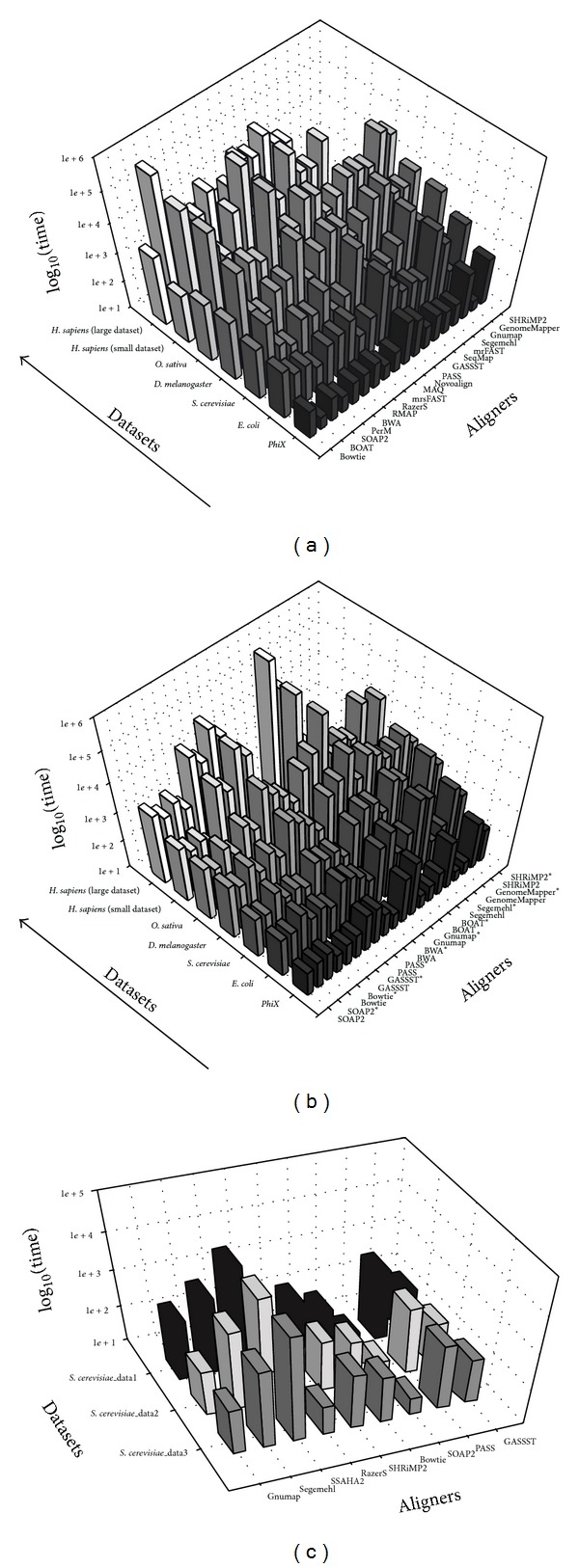
Bar graph illustrates a comparison of different computation time plots against multiple aligners. In this Figure, *z*-axis is log value of the computation time, *y*-axis represents real-life datasets, and *x*-axis represents multiple aligners under this comparison. Based on real-life short-read datasets sampled from various organisms by Illumina sequencing platform, (a) displays computation time comparison in single-thread mode, (b) displays computation time comparison for both in single-thread mode and in three-thread mode, and (c) displays computation time comparison in single-thread mode based on real-life long-read datasets by Roche 454 sequencing platform. (∗) represents the results for aligners supported multiple threads function evaluated in three-thread mode.

As the results shown in Figure 2(a), computation time is plotted against the favorable multiple aligners. The short-read datasets sampled from various organisms, namely, virus* PhiX174*, bacteria* Escherichia coli,* yeast* Saccharomyces cerevisiae*, fruit fly* Drosophila melanogaster*, plant* Oryza sativa*, and human* Homo sapiens*, were used to assess the impact of reference genome size on computation time. Clearly, most of aligners showed a linear relationship between the computation time and the size of reference genome. Besides the genome size, the count of reads had impact on computation time as clearly seen from 2 short-read datasets of* Homo sapiens* with different read counts. Noticeably, it should be stressed that computation time of Novoalign showed more dependence on the count of reads than reference genome size. The detailed information for real-life short-read datasets and reference genomes was listed in Table 2. In such a case of comparison between plant genome (i.e.,* O. sativa*) and human genome (i.e.,* H. sapiens*), we observed that the computation time of plant genome (>5 hours) was slower than human genome (1.5 hours).

From overall results with short-read datasets produced by Illumina sequencing platform as shown in Figures 2(a) and 2(b), we observe that the computation speed for Bowtie, SOAP2, BWA, and PerM was significantly faster than the other aligners regardless of different reference genome sizes and read counts. These results may be explained by BWT-based backtracking algorithm behind Bowtie, SOAP2, and BWA which probably impacted on reduction of computation time. In particular, PerM obviously showed an outstanding computation speed due to simultaneous utility of available multiple threads. On the other hand, BOAT and RazerS required significant amounts of computation time. Their computation speed was extremely slower than the others under the same computational conditions (see Section 4). Once multiple threads are utilized, computation speed was dramatically increased, such as BOAT (see Figure 2(b)). For the other aligners, apart from Segemehl, Gnumap, and SHRiMP2 [30], the major of aligners obtained ideal computation speed during small reference genome analysis process (e.g., virus, bacteria, etc.). With multiple threads utilized, computation time of the aligners was significantly reduced, such as PASS, GASSST, SHRiMP2, and Segemehl. The results are shown in Figure 2(b). In addition, Figure 2(c) shows a plot of computation time against multiple aligners, regarding long-read datasets generated by Roche 454 sequencing platform sampled from yeast* S. cerevisiae*. The detailed information for real-life long-read datasets was listed in Table 3. We observed that SSAHA2, Segemehl, and PASS required significant amounts of computation time; in contrast to Bowtie, SOAP2, RazerS, and GASSST relatively showed high computation speed.

#### 2.3.2. Maximum Memory Usage Comparison

**Figure 3:**
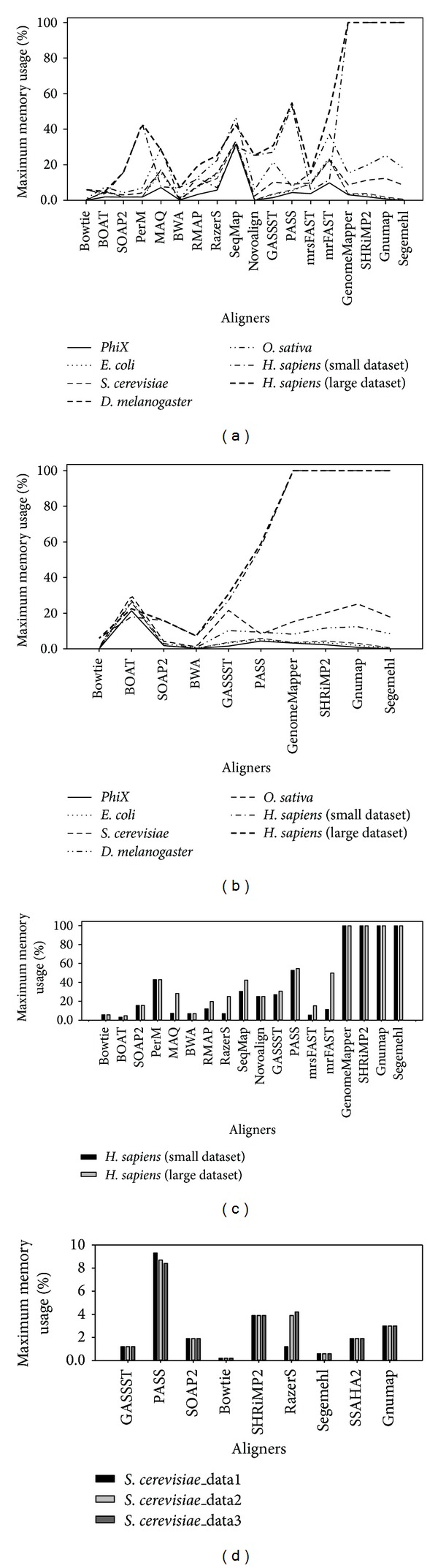
Graphical representation shows a comparison of various memory usage plots against multiple aligners. With real-life short-read datasets sampled from various organisms by Illumina sequencing platform, (a) shows the memory usage requirements of multiple aligners in single-thread mode, (b) shows the memory usage requirements of multiple aligners in three-thread mode, and (c) shows correlations among read count, genome size, and memory usage. Two short-read datasets (e.g., 5 million reads and 18 million reads) from* H. sapiens* were chosen to perform comparative analysis. In addition, (d) shows the memory usage requirements of multiple aligners with real-life long-read datasets produced by Roche 454 platform.

For memory usage comparison, we quantified variation of maximum memory usage by cross-comparisons among multiple aligners against maximum memory usage percentage (%) of the server. As illustrated in Figure 3(a), several bottom spots in the plot are clearly pointed out to represent the aligners with relatively minor memory usage during short-read datasets aligning process, which were Bowtie, BOAT, SOAP2, BWA, and mrsFAST. The maximum memory usage occupations of these aligners were relatively low and not dependent on the genome size analyzed. It was clearly seen in analysis of human genome as a reference that the maximum memory usage percentages of these aligners were 6.0%, 4.9%, 15.8%, 7.2%, and 15.4%, respectively. Thus, if even low hardware capacity was used, these aligners could not be any problem and could run with full usage on the PC computers. Yet, BOAT had dramatically increased in memory usage when multiple threads were applied (see Figure 3(b)). These results may be explained from the root of data structure constructed, such as bitmap index and prefix tree data structure in BOAT. For PerM, Novoalign, GASSST, and PASS, low memory usage was occupied with small reference genome analyzed, but a sharp increase in memory usage appeared with human genome analyzed in comparison with others, namely, 43.0%, 25.3%, 30.8%, and 54.7%, respectively. Moreover, in case of human genome analyzed, memory usages of GenomeMapper, SHRiMP2, Gnumap, and Segemehl were out of the limitation of the servers.

In addition, we found that maximum memory usage of majority of aligners was kept stable with multiple threads function employed excluding BOAT, PASS, and SHRiMP2. In particular in BOAT, it was slightly shown to be increased in memory usage (Figure 3(b)). Because of the differences in alignment algorithms constructing the index of reads, these greatly made influences on memory usage occupation. This is shown in Figure 3(c). Hence, it is apparently illustrated that the aligners, such as BOAT, MAQ, RMAP, RazerS, SeqMap, mrFAST, and mrsFAST, showed variable memory requirements mainly depending on the count of the reads instead of size of genome, while the aligners, including Bowtie, SOAP2, BWA, PerM, Novoalign, PASS, and GASSST, showed constant memory requirements regardless of the count of reads. Besides, Figure 3(d) shows comparison of the maximum memory usage of different aligners under the long-read datasets from Roche 454 sequencing platform. It was further confirmed that SOAP2, Bowtie, SHRiMP2, and Segemehl showed constant memory requirements regardless of the count of reads and the type of reads as well. Moreover, PASS seemed to show relatively higher requirement for memory usage when it deal with long-read datasets.

#### 2.3.3. Mapped Read Counts Comparison

**Figure 4:**
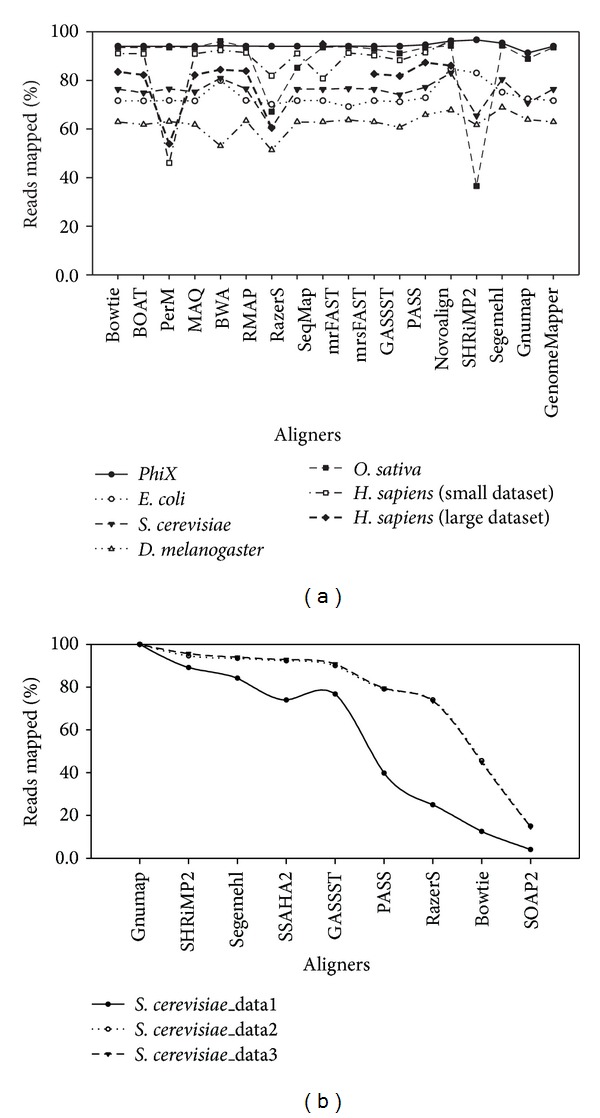
Graphical representation shows a comparison for different mapped reads count plots against multiple aligners with real-life short-read datasets and long-read datasets, respectively.

For mapped read counts, it is considered to be another key factor for computational performance evaluation, since it can quantify relative read density. We calculated the mapped read counts across different aligners. As shown in Figure 4(a), we observe that most aligners showed very similar results of mapped read counts excepting SOAP2, RMAP, and SHRiMP2 which represented low percentage of mapped read counts with the short-read datasets used. On the other hand, we compared the results of mapped read counts with long-read datasets as well (Figure 4(b)). It was clearly shown that SHRiMP2, Segemehl, GASSST, SSAHA2, and Gnumap had relatively better results compared with the rest of aligners. However, we could not make a judgment for capability and sensitivity of mapping aligners, since real-life data could not be employed to evaluate alignment accuracy. Further comparative analysis with *in silico * data is described in following.

#### 2.3.4. Alignment Accuracy Evaluation Using* In Silico* Datasets

In order to evaluate alignment accuracy of individual aligner, we calculated sensitivity, precision, and % of multimapped reads as indicator values for evaluation. Moreover, we took mismatches, indels, and read lengths into consideration during aligning and mapping process.

**Figure 5:**
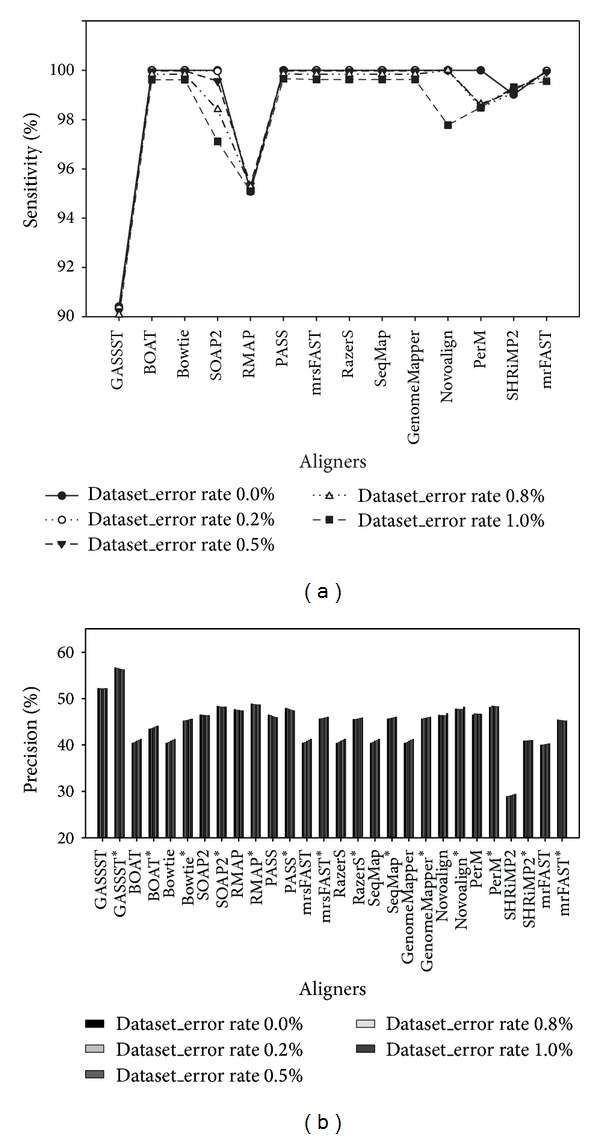
Graphical representation shows alignment accuracy results using* in silico* short-read datasets with varying error rates. Based on* in silico* short-read datasets sampled from chromosome X of* H. sapiens* with varying error rates (e.g., 0%, 0.2%, 0.5%, 0.8%, and 1.0%, resp.), (a) and (b) show accuracy evaluation by sensitivity and precision, respectively. Aligners with (∗) in (b) are used to show alignment accuracy evaluation by precision with consideration of multimapped reads.

To indicate alignment accuracy evaluation for short-read datasets with varying error rate existing, the results are shown in Figure 5(a) for sensitivity and Figure 5(b) for precision comparison. We could see that most aligners showed relatively high sensitivity over 98%, excluding RMAP and GASSST. For Bowtie, Novoalign, and PerM, their sensitivity significantly decreased as the error rate increased (Figure 5(a)). Furthermore, Figure 5(b) also shows that GASSST possessed outstanding performance for precision comparison and PerM, Novoalign, PASS, RMAP, and SOAP2 presented the same level of precision followed behind GASSST, without consideration of multimapped reads. It was also noticed that SHRiMP2 had weak performance in terms of precision. With consideration of multimapped reads, most aligners, excluding PerM, Novoalign, PASS, RMAP, and SOAP, were slightly increased in precision, especially SHRiMP2.

**Figure 6:**
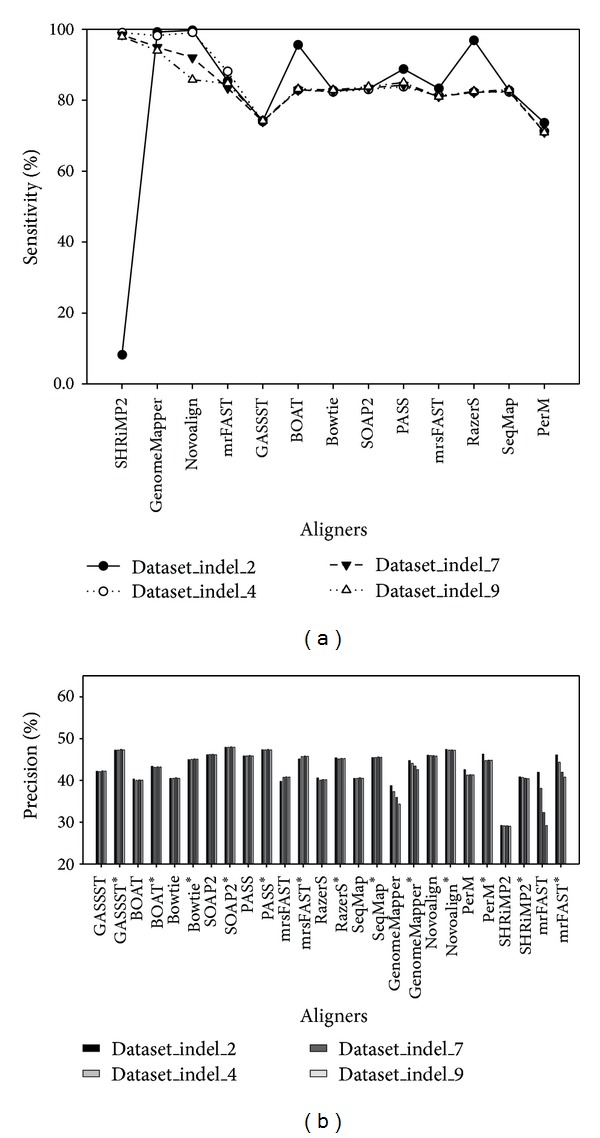
Graphical representation shows alignment accuracy results using* in silico* short-read datasets with varying indel sizes. Based on* in silico* short-read datasets sampled from chromosome X of* H. sapiens* with varying indel sizes (e.g., 2 bp, 4 bp, 7 bp, and 9 bp, resp.), (a) shows alignment accuracy evaluation by sensitivity and (b) shows alignment accuracy evaluation by precision. Aligners with (∗) as shown in (b) are used to show alignment accuracy evaluation by precision with consideration of multimapped reads.

As expected, Figure 6 shows alignment accuracy evaluation for short-read datasets with fixed indel frequency (0.1%) as the average indel sizes vary. Apparently, we found that GASSST and PerM were confirmed to have weak performance in sensitivity (<80%), but SHRiMP2, GenomeMapper, and Novoalign had relatively high sensitivity from overall results (Figure 6(a)). In addition, it can be seen in Figure 6(b) that Novoalign, PASS, SOAP2, and GASSST showed very favorable precision values, while SHRiMP2 provided the unsatisfactory precision value. However, it can also been seen that precision was improved by almost 5% among GASSST, mrsFAST, mrFAST, RazerS, SeqMap, GenomeMapper, and SHRiMP2, when multimapped reads were considered. Meanwhile, it was emphasized that GenomeMapper and mrFAST might not be better suited for indel calling due to their weak accuracy in terms of both sensitivity and precision, as indel sizes significantly increased.

**Figure 7:**
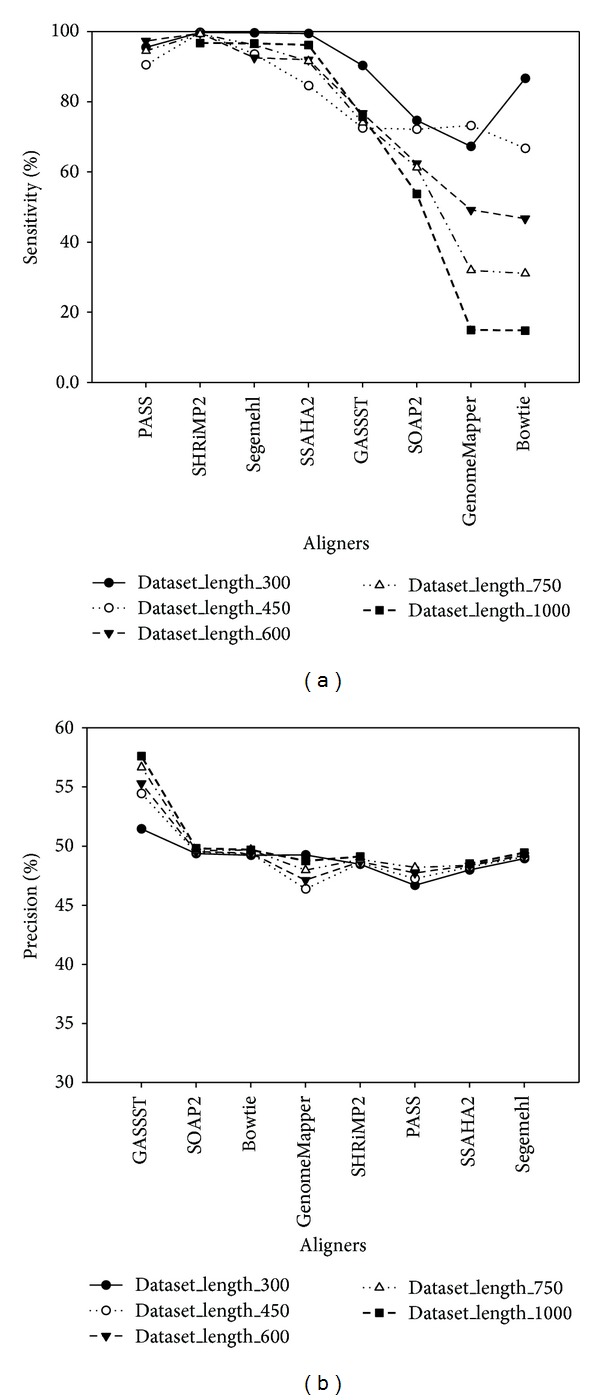
Graphical representation shows alignment accuracy results using* in silico* long-read datasets with varying read lengths. Based on* in silico* long-read datasets sampled from* E. coli* at different lengths of 300 bp, 450 bp, 600 bp, 750 bp, and 1000 bp, evaluated by 8 aligners (e.g., GASSST, Bowtie, SOAP2, PASS, SSAHA, SHRiMP2, GenomeMapper, and Segemehl), (a) shows alignment accuracy evaluation by sensitivity and (b) shows alignment accuracy evaluation by precision.

The alignment accuracy evaluation provided by multiple aligners supported long-read alignment with varying read length on* E. coli* genome was primarily highlighted in Figure 7. As seen in this figure, PASS, SHRiMP2, Segemehl, and SSAHA2 had the highest sensitivity, while SOAP2, GenomeMapper, and Bowtie presented relatively low sensitivity and their sensitivity depended strictly on read length (Figure 7(a)). Moreover, it is also clearly seen in Figure 7(b) that GASSST showed the highest sensitivity and a significant increase in sensitivity with increasing read lengths. It seems that GASSST was the most robust to longer reads and particularly useful as reads get longer.

**Figure 8:**
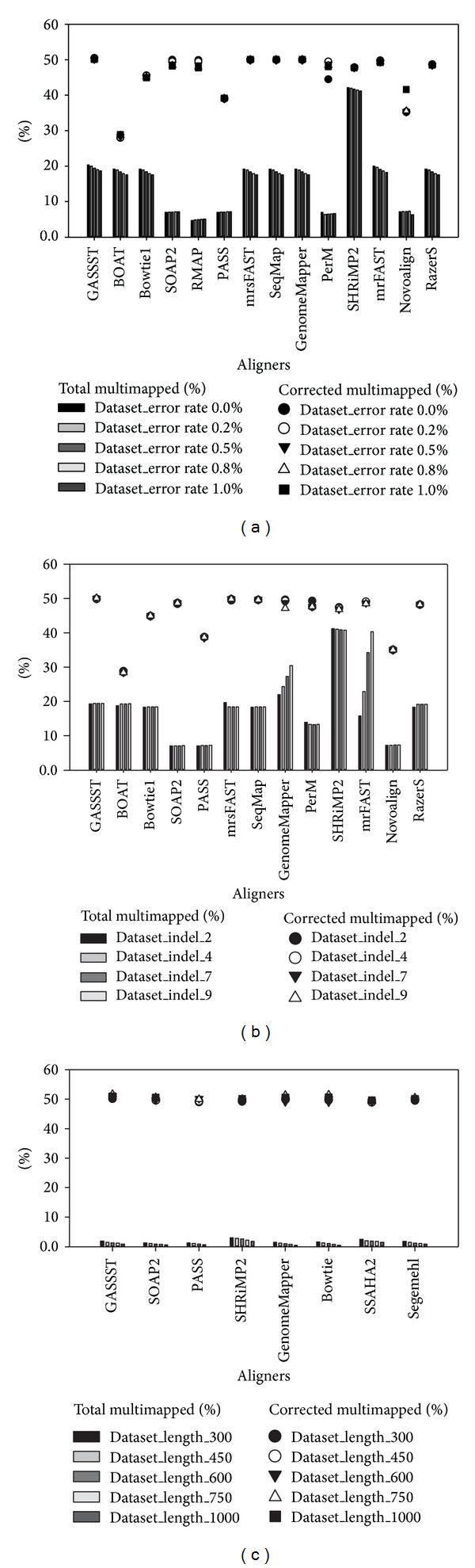
Graphical representation shows impact of total multimapped reads and corrected multimapped reads on alignment accuracy results using* in silico* datasets. (a), (b), and (c) show % of total multimapped reads and % of corrected multimapped reads for* in silico* datasets with varying error rates, indel sizes, and read lengths, respectively.

For datasets with varying error rates, indel sizes and read lengths existed; the results are shown in Figure 8. We evaluated % of total multimapped reads and % of corrected multimapped reads. As presented in Figure 8, the results were used to confirm influence of multimapped reads on alignment accuracy. GASSST, SHRiMP2, GenomeMapper, SeqMap, RazerS, mrFAST, mrsFAST, Bowtie, and BOAT could provide relatively high percentage of total multimapped reads (>20%) and high percentage of corrected multimapped reads as well when dealing with short-read datasets with varying error rates and indel sizes, especially SHRiMP2 (Figures 8(a) and 8(b)). However, it was indicated that these aligners could provide more information within multimapped reads, and this might result in missing important biological information without consideration of multimapped reads. In contrast, when dealing with long-read datasets with varying read lengths, the situation showed a tremendous difference in percentage of total multimapped reads and correctly mapped multimapped reads. Less information was provided by all the aligners within multimapped reads for long-read aligning and mapping. The results are shown in Figure 8(c).

## 3. Conclusions

Currently, optimal aligners have been called for the variety of applications and specific types of data-based NGS technology. This study aims to systematically evaluate and compare the capability of multiple aligners to provide guiding resource for choosing suitable aligners dependent on the user's specific research aims with NGS data. We evaluated multiple aligners based on criteria, including application-specific alignment feature, computational efficiency, and alignment accuracy. To assess the multiple aligners, real-life short-read datasets and long-read datasets sampled from various organisms and* in silico* datasets with varying error rates, indel sizes, and read lengths were considered as standard datasets for different applications and sequencing technologies.  Table 4 provided the overall summary on aligning and mapping evaluations in terms of computation speed, memory usage, and accuracy as well. It is concluded that Bowtie, BWA, and SOAP2 clearly show high computational efficiency in single-thread mode and increasing trend of computation efficiency in multi-thread mode on real-life datasets. However, PerM and Novoalign show outstanding performance on improving computation efficiency by adjusting thread mode automatically and indexing read datasets, respectively. Indeed we conclude that they can be suitable and efficient aligners for short-read aligning and mapping. It is also shown that memory usage requirements of Bowtie, BWA, BOAT, mrsFAST, and SOAP2 are relatively low both in single-thread mode and multithread mode and their memory usage requirements are kept low regardless of the number of reads and the size of genomes. Moreover, it could be seen that GenomeMapper, Novoalign, and SHRiMP2 show high sensitivity, while GASSST, Novoalign, PASS, and SOAP2 show high precision when dealing with mismatch and indel errors existed in simulated datasets. With high alignment accuracy evaluation obtained from* in silico* datasets, we conclude that GASSST, PerM, Novoalign, PASS, RMAP, and SOAP2 can be better choices, since they possess high accuracy without indels for ungapped alignment, while Novoalign, PASS, and SOAP2 have high accuracy with indels for gapped alignment. In particular, GASSST can be a candidate aligner for long reads aligning and mapping. In addition, it is implied that Novoalign and Segemehl can be representative aligners to apply for wide applications, such as gapped alignment for SNPs and structural variation discovery, paired-end alignment for mapping of repetitive region, bisulfite alignment for ChIP sequencing data analysis, and SNPs calling. Finally, we believe that our evaluation will be a benefit for biologists engaged in variety of genomics researches. The overall evaluation and comparison of multiple aligners for NGS data analysis might serve as an essential recommendation for suitable selection of aligners.

## 4. Methods

**Figure 9:**
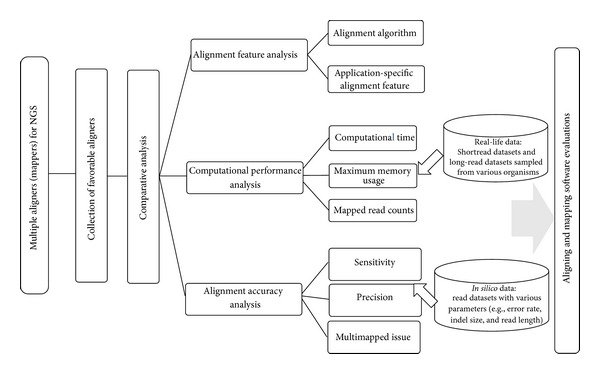
Flow chart for evaluation and comparison process of multiple aligners. The process contains three main steps, namely, alignment feature comparison, computational performance comparison, and alignment accuracy comparison for NGS data analysis.

The pipeline of the whole procedure in this study is illustrated in Figure 9. We collected 25 unspliced read aligners developed for NGS data from different websites and published articles (Supplementary File 1). Notably, spliced read aligners were not taken in this evaluation and comparison because they were primarily used to map the reads from exon-exon junctions, which were specific algorithm for RNA-Seq [43]. However, the aligners with any extra mandatory, which made them unavailable for most of biologists, were not taken into account. For example, SOAP3 [44] depended on a CUDA-enabled GPU, CloudBurst [45] required cloud computing, and ZOOM [33] was commercial version. Therefore, 19 favorable aligners were eventually considered for further evaluation and comparison process. Details of the selected aligners are shown in Supplementary File 2.   Supplementary File 3 shows the number of citation papers associated with each aligner in order to provide the information of the popularity. In the following, we describe evaluation and comparison of the multiple aligners.

### 4.1. Evaluation and Comparison of the Multiple Aligners

#### 4.1.1. Literature Searching and Programming Implementation for Application-Specific Alignment Features Evaluation

To evaluate application-specific alignment features, at the beginning, we performed literature searching to grasp and compare the alignment algorithms of 19 favorable aligners. Based on principal common characteristics of alignment algorithms sharing by multiple aligners, we then classified these aligners into two different algorithms applied, namely, hash table-based algorithm and BWT-based backtracking algorithm. However, information about important alignment features or characteristics of the multiple aligners is essential for various genomewide association studies. To collect and evaluate application-specific alignment features, we manually mined literature and other documentation and inspected the source code for individual aligner. Moreover, we implemented our own programming for individual aligner according to its alignment features as well. The application-specific alignment features were considered as follows: multithread, gapped alignment analysis, paired-end alignment analysis, trimming alignment analysis, and bisulfite alignment analysis.

#### 4.1.2. Using Real-Life Data for Accessing Computational Performance

To evaluate computational performance for different practical applications, we used 3 real-life long-read datasets from Roche 454 sequencing platform and 7 real-life short-read datasets from Illumina sequencing platform as representative input. They were sampled from various organisms, namely, virus* PhiX174* (1 dataset), bacteria* Escherichia coli* (1 dataset), yeast* Saccharomyces cerevisiae* (4 datasets),* fruit fly Drosophila melanogaster* (1 dataset), plant* Oryza sativa* (1 dataset), and human* Homo sapiens* (2 datasets). They were downloaded from National Center for Biotechnology Information (NCBI) Short-Read Archive (http://www.ncbi.nlm.nih.gov/Traces/sra/). In addition, the reference genome sequences were obtained from NCBI (http://www.ncbi.nlm.nih.gov/) and UCSC Genome Browser Home (http://genome.ucsc.edu/). The description of real-life datasets from different sequencing platform was detailed in Tables 2 and 3.

Besides input data used for evaluation, computer hardware requirements and determined parameters setting were also concerned. For computer hardware platform, we used a large-memory server with a four-core 2.4 GHzAMD Opteron processor and a maximum of 32 GB of RAM. For parameters setting, two mismatches were allowed within a full read length without considering any insertions and deletions (indels) during the mapping process of Illumina short-read datasets, while gapped alignment was allowed considering indels during the mapping process of Roche 454 long-read datasets, since indel frequency is extremely low within short-read datasets produced by Illumina sequencing platform instead of long-read datasets produced by Roche 454 sequencing platform. In addition to the default parameter values, the other parameters for each aligner were applied in an attempt to achieve parameter optimization. To account for threading when assessing computational efficiency, we employed all the aligners to perform aligning process in single-thread mode without any competition and we also were careful about some aligners supported multiple threads function to accelerate computation speed; thus these aligners were evaluated and compared in three-thread mode without any competition.

Computational performance was evaluated by consideration of three factors: computation time, maximum memory usage, and mapped read counts. These three factors mainly used to measure computational efficiency, hardware availability, and qualified read density. To obtain computation time, wall-clock time was computed for each computational process with excluding index time. Since computation time was slightly affected by computational condition of the hardware, minor discrepancy appeared definitely during each computational process. Thus, we chose the set of results under relatively stable computational process as representative results across multiple runs.

To record maximum memory usage, we developed a tool written by Python (Supplementary File 4) to monitor each programming process and then reported maximum memory usage percentage of our server's memory (32G). For mapped read counts, not only we considered uniquely mapped reads but also multimapped reads were included in the mapped reads to provide a rough perspective of alignment sensitivity for each aligner.

#### 4.1.3. Using* In Silico* Data for Accessing Alignment Accuracy

To access capability of individual aligner, we evaluated not only computational performance but also alignment accuracy. It has limitations to use real-life data for accessing alignment accuracy, since true alignment locations are unknown. Hereby, we therefore wrote a Perl script to generate* in silico* data by computational simulation (Supplementary File 5). Concerning influence of mismatches, indels, and read lengths,* in silico* datasets were therefore generated according to the characteristics as listed in Table 5. The characteristics included read lengths, read counts, sequencing error rates, indel sizes, and indel frequency. Once the simulating completed, 9* in silico* short-read datasets from chromosome *X* of* H. sapiens* were achieved. In addition to short-read datasets, we also simulated 5 long-read datasets from* E. coli* with different lengths. Besides* in silico* data, computer hardware requirements were similarly determined as previously described for accessing computational performance section. Exceptionally during the mapping process, parameters (e.g., maximum allowed mismatches and indels) were set upon own datasets feature. Finally, we measured the alignment accuracy of different aligners in terms of sensitivity and precision. The formula is shown as follows:

(1) $$\begin{array}{c} \text{Sensitivity}=\frac{\text{TP}}{\text{FP}+\text{FN}}\text{Precision}=\frac{\text{TP}}{\text{TP}+\text{FP}}. \end{array}$$

In addition, we further took multimapped reads into consideration, which were ambiguously mapped. Multimapped reads existing in alignment results frequently cause difficulty for the biologists to choose their real locations. This may result in missing some biological information. Thus, % of multimapped and % corrected of multimapped are thus applied as new criteria to access the capability of these aligners as follows:

(2) %  Total  multi-mapped  reads  =multimapped  readsmultimapped  reads+unique  mapped  reads,%  Corrected  multimapped  reads  =Corrected  multimapped  readsmultimapped  reads.

## Supplementary Material

The supplementary materials include the detail information for 25 aligners collected from different websites and publication (Supplementary file 1), the list of the aligners involved in the evaluation and comparison process (Supplementary file 2), numbers of citations for multiple aligners reflecting their popularity (Supplementary file 3), a python script used as memory usage monitor (Supplementary file 4) and a Perl script for computational simulation of in silico data (Supplementary file 5).

## Figures and Tables

**Table 1:** Application-specific alignment features distribution among multiple aligners.

Aligners	Operate system	Programming language	Input Format^1^? (Fasta and Fastq)	Output format	Multithread?	Gapped alignment?	Paired-end alignment?	Trimming alignment?	Bisulfite alignment?	Note
Bowtie	★	C++	*√*	SAM	*√*		*√*	*√*		Maximum allowed mismatches ≤3
BWA	⊚	C++	*√*	SAM	*√*	*√*	*√*			BWA-short: 200 bp; BWA-SW: 100 kbp
BOAT	⊚	C	*√*	∗	*√*	*√*				Maximum allowed mismatches ≤3
GASSST	⊚	C++	Fasta	SAM	*√*	*√*				Merely Fasta format required for reads
Gnumap	⊚	C	*√* ( prb)	SAM	*√*	*√*		*√*	*√*	Maximum read length <1000 bp
GenomeMapper	⊚	C	*√*	BED	*√*	*√*				Maximum read length < 2000 bp
mrFAST	★	C	*√*	SAM		*√*	*√*			Maximum read length <300 bp
mrsFAST	★	C	*√*	SAM			*√*		*√*	Maximum read length <200 bp
MAQ	⊚	C++	Fastq	map			*√*			Maximum read length ≤128 bp
NovoAlign	●	C++	*√*	SAM	*√*	*√*	*√*	*√*	*√*	Restrictions for academic version
PASS	*※*	C++	*√* ( sff )	GFF3	*√*	*√*	*√*			Maximum read length <1000 bp
PerM	*※*	C++	*√*	SAM	*√*		*√*	*√*		Maximum read length ≤128 bp
RazerS	★	C++	*√* ( prb)	Eland, GFF		*√*	*√*	*√*		Arbitrary read length
RMAP	⊚	C++	*√*	BED			*√*		*√*	Fixed-length reads required
SeqMap	★	C++	Fasta	Eland		*√*				Maximum allowed mismatches ≤5
SOAPv2	⊚	C++	*√*	∗	*√*	*√*	*√*			Maximum read length <1000 bp
SHRiMAP2	⊚	Python	Fasta	SAM	*√*	*√*	*√*			Parallel computing supported
Segemehl	⊚	C	Fasta	∗	*√*	*√*	*√*	*√*	*√*	Large memory usage required
SSAHA2	●	NA	*√*	GFF, SAM			*√*			For long reads mapping

^1^We here only consider short-reads input format.

^*※*^Windows, Linux, or Unix operating system.

^★^Windows, Linux,Unix, or Mac X operating system.

^●^Linux,Unix, or Mac X operating system.

^⊚^Linux or Unix operating system.

*The short-read aligning algorithms' own output format.

**Table 2:** Detailed information for reference genomes and real-life short-read datasets from Illumina sequencing platform.

Genome	Reads ID	Reads length (bp)	Read count	Genome size	Genome version (ID)
*PhiX *	ERR007488	36	4516934	<1 Mbp	NC_001422.1 (NCBI)
*E*. *coli *	SRR023978	51	9575373	5 Mbp	NC_000913.2 (NCBI)
*S*. *cerevisiae *	SRX011891	36	10995605	12 Mbp	sacCer2 (UCSC)
*D*. *melanogaster *	SRR001815	36	10760364	172 Mbp	dm3 (UCSC)
*Oryza sativa *	DRR000023	32	18443432	388 Mbp	NCBI
*Homo sapiens *	SRR037152	35	4761769	3263 Mbp	hg18 (UCSC)
*Homo sapiens *	SRX003935	32	18424533	3263 Mbp	hg18 (UCSC)

**Table 3:** Information for reference genomes and real-life long-read datasets from Roche 454 platform.

Genome	Reads ID	Read length (bp)	Read count	Genome size	Genome version (ID)
*S*. *cerevisiae *	SRR001091	100–200	323986	12 Mbp	sacCer2 (UCSC)
*S*. *cerevisiae *	SRR001092	100–200	409212	12 Mbp	sacCer2 (UCSC)
*S*. *cerevisiae *	SRR001093	100–200	430794	12 Mbp	sacCer2 (UCSC)

**Table 4:** Overall evaluation and comparison of multiple aligners.

Aligners	Computational speed			Memory usage			Accuracy			
	Speed with single thread	Speed with multithread	Key factor impacting speed (genome size or read count)	Overall evaluation	Key factor impacting memory (Genome size or read count)	Memory usage with multithread	Sensitivity	Precision	% of multimapped	%Corrected Multi-Mapped
Bowtie1	Fast	↑	Genome size	Low	Genome size	*≡*	High	—	—	
BWA	Fast	↑	Both	Low	Genome size	*≡*				
BOAT	Slow	↑↑	Genome size	Low	Read count	↑↑	High	—	—	Low
GASSST	—	↑	Genome size	High^★★^	Genome size	*≡*	Low	High	—	
Gnumap	Slow	↓	Genome size	High^★★^	Genome size	*≡*				
GenomeMapper	Slow	*≡*	Genome size	Low^▲^	Genome size	*≡*	High	—	—	
mrFAST	Slow	×	Genome size	High^★★^	Read count	×	High	—	—	
mrsFAST	—	×	Genome size	Low	Read count	×	High	—	—	
MAQ	—	×	Genome size	High^★★^	Read count	×				
NovoAlign^#^	—	/	Read count	Low^▲^	Genome size	/	High	High	Low	Low
PASS	—	↑	Genome size	Low^▲^	Genome size	↑	High	High	Low	Low
PerM^*※*^	Fast		Genome size	Low^▲^	Genome size	/	Ind: low	—	Low	
RazerS	Slow	×	Genome size	High^★★^	Read count	×	High	—	—	
RMAP	—	×	Genome size	High^★^	Genome size	×	Mis: low	High	Low	
SeqMap	—	×	Genome size	High^★★★^	Read count	×	High	—	—	
SOAPv2	Fast	↑	Genome size	Low	Genome size	*≡*	High	High	Low	
SHRiMAP2	Slow	↑	Genome size	High^★★^	Genome size	↑	High	Low	High	
Segemehl	—	↑	Both	High^★★★^	Genome size	*≡*	High	—		

PerM^*※*^ could adjust the threads automatically during running process.

Novoalign^#^ could support multithread only for commercial version.

For computational speed, we defined the aligners which are extremely faster than others as fast, while we defined the ones which are extremely slower as slow.

For memory usage, we evaluated the aligners as follow: among the s even datasets, the maximum memory usage ≤4 G, low; the maximum memory usage ≥32 G, high^★★★^.

Low^▲^ represents that the maximum memory usage will have an extreme increase with *H*. *sapiens* datasets (≥4 G).

×: without multithread function.

— represents medium level remark.

*≡* means there is no obvious change.

**Table 5:** Parameters setting for *in silico* data: read lengths, error rates, indel sizes, indel freq.

Accuracy Evaluation	Read length (bp)	Read number	Error rate (%)	Indel size (bp)	Indel freq. (%)
Mismatch factor	50	5000000	0, 0.2, 0.5, 0.8, 1.0	0	0
Indel factor	50	5000000	0	2, 4, 7, 9	0.1
Read-length factor	300, 450, 600, 750, 1000	1000000	0.5	4	0.1
