# Supplementary material for: Evaluation and Comparison of Multiple Aligners for Next-Generation Sequencing Data Analysis
Source: Biomed Res Int. 2014 Mar 23;2014:309650. doi: 10.1155/2014/309650 (PMC3980841; doi:10.1155/2014/309650)
Supplement: Supplementary file 1 — The supplementary materials include the detail information for 25 aligners collected from different websites and publication (Supplementary file 1), the list of the aligners involved in the evaluation and comparison process (Supplementary file 2), numbers of citations for multiple aligners reflecting their popularity (Supplementary file 3), a python script used as memory usage monitor (Supplementary file 4) and a Perl script for computational simulation of in silico data (Supplementary file 5). [file 309650.f1.pdf]

Supplementary file 1.The detail information for 25 aligners collected from different websites and published articles

Software	OS	Language	Reference	Links
**BOAT**	Linux/Unix 32/64bit	C	PMID: 19958483	http://boat.cbi.pku.edu.cn
**Bowtie**	Linux/Unix 32/64bit<br>Windows<br>Mac OS X/Solaris	C++	PMID:19261174	http://bowtie-bio.sourceforge.net/index.shtml
**BS Seeker**	Linux/Unix 32/64bit<br>Mac OS X 32/64bit	Python	PMID:20416082	http://sourceforge.net/projects/bsseeker/files/BS%20Seeker2004-23-2010/examples.tgz/download
**BWA**	Linux/Unix 32/64bit	C++	PMID:19451168	http://bio-bwa.sourceforge.net/
**GASSST**	Linux/Unix 32/64bit	C++	PMID: 2073910	http://www.irisa.fr/symbiose/projects/gassst/
**GenomeMapper**	Linux/Unix 32/64bit	C	PMID:19761611	http://1001genomes.org/downloads/genomemapper.html
**GSNAP**	Linux/Unix 32/64bit	C	PMID: 20147302	http://research-pub.gene.com/gmap/
**GNUMAP**	Linux/Unix 32/64bit	C	PMID:19861355	http://dna.cs.byu.edu/gnumap/download.cgi

Supplementary file 2. The version list of the aligners involved in the evaluation process

Software	Version	websites
**Bowtie**	bowtie-v0.12.7	http://bowtie-bio.sourceforge.net/index.shtml
**SOAPv2**	SOAPaligner-v2.21	http://soap.genomics.org.cn/
**BWA**	bwa-0.5.8a	http://bio-bwa.sourceforge.net/
**PerM**	PerM-v0.3.6	http://code.google.com/p/perm/
**mrsFAST**	mrsfast-v2.3.0.2	http://sourceforge.net/projects/mrfast/files/mrsfast/2.3.0.2/
**GASSST**	Gassst-v1.28	http://www.irisa.fr/symbiose/projects/gassst/
**PASS**	pass-v1.62	http://pass.cribi.unipd.it/cgi-bin/pass.pl
**BOAT**	boat-v1.0	http://www.seqan.de/downloads/projects.html#c13
**RazerS**	RazerS-20100618	http://bowtie-bio.sourceforge.net/index.shtml
**RMAP**	rmap-v2.05	http://rulai.cshl.edu/rmap/
**MAQ**	maq-0.7.1	http://maq.sourceforge.net/
**Novoalign**	Novocraft-v2.05	http://www.novocraft.com/
**mrFAST**	mrFast-v2.1.0.1	http://mrfast.sourceforge.net/
**SeqMap**	SeqMap-v 1.0.12	http://biogibbs.stanford.edu/~jiangh/seqmap/
**Segemehl**	Segemehl-v0.1.2	http://www.bioinf.uni-leipzig.de/Software/segemehl/
**SHRiMAP**	SHRiMP-v2.2.1	http://compbio.cs.toronto.edu/shrimp/
**Gnumap**	Gnumap-v3.0.2	http://dna.cs.byu.edu/gnumap/
**GenomeMapper**	genomemapper0.4.3	http://1001genomes.org/downloads/genomemapper.html
**SSAHA2**	ssaha2_v2.5.3	http://www.sanger.ac.uk/resources/software/ssaha2/

**MAQ**	Linux/Unix 32/64bit	C++	PMID:18714091	http://maq.sourceforge.net/
**MOM**	Windows 32/64bit Linux/Unix 32/64bit	Java	PMID: 19228804	http://mom.csbc.vcu.edu/
**Mosaik**	Windows 32/64bit Linux/Unix 32/64bit	C++	N/A	http://bioinformatics.bc.edu/marthlab/Mosaik
**mrFAST & mrsFAST**	Windows 32/64bit Linux/Unix 32/64bit	C	PMID: 19718026 PMID: 19447966 PMID: 19483690	http://mrfast.sourceforge.net/
**MUMmer**	Linux/Unix 32/64bit	C++	PMID: 14759262	http://mummer.sourceforge.net/
**Novoalign**	Linux/Unix 32/64bit	C++	N/A	http://www.novocraft.com/
**PASS**	Windows 32/64bit Linux/Unix 32/64bit	C++	PMID:19218350	http://pass.cribi.unipd.it/cgi-bin/pass.pl
**PerM**	Windows 32/64bit Linux/Unix 32/64bit	C++	PMID: 19675096	http://code.google.com/p/perm/
**RazerS**	Windows 32/64bit Linux/Unix 32/64bit Mac X OS	C++	PMID: 19592482	http://www.seqan.de/projects/razers.html

	32/64bit			
**RMAP**	Linux/Unix<br>32/64bit	C++	PMID:19736251	http://rulai.cshl.edu/rmap/
**Segemehl**	Linux/Unix<br>32/64bit	C	PMID:19750212	http://www.bioinf.uni-leipzig.de/Software/segemehl/
**SeqMap**	Windows<br>32/64bit<br>Linux/Unix<br>32/64bit<br>Mac X OS<br>32/64bit	C++	PMID: 18697769	http://biogibbs.stanford.edu/~jiangh/seqmap/
**SHRiMP**	Linux/Unix<br>32/64bit	Python	PMID: 19461883	http://compbio.cs.toronto.edu/shrimp/
**SliderII**	Windows<br>32/64bit<br>Linux/Unix<br>32/64bit<br>Mac X OS<br>32/64bit	Java	PMID: 18974170	http://www.bcgsc.ca/platform/bioinfo/software/SliderII
**SOAP2**	Linux/Unix<br>64bit	C++	PMID: 18227114<br>PMID: 19497933	http://soap.genomics.org.cn/
**SSAHA2**	Linux/Unix<br>32/64bit<br>Mac X OS<br>32/64bit	N/A	PMID: 11591649	http://www.sanger.ac.uk/resources/software/ssaha2.html
**BFAST**	Linux/Unix<br>32/64bit	Python	PMID: 19508732<br>PMID: 19907642	https://sourceforge.net/apps/mediawiki/bfast/index.php?title=Main_Page

Supplementary file 3. The numbers of citation papers for multiple aligners

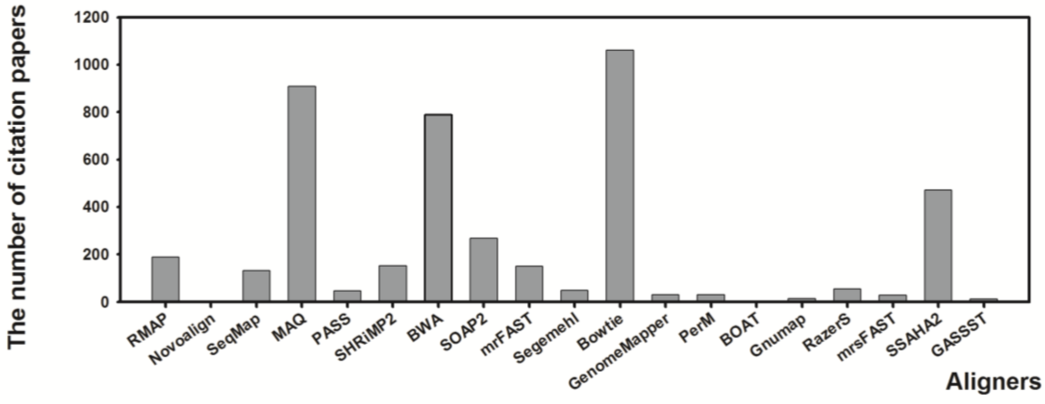

Supplementary file 4. A python script was mainly used as memory usage monitor

```
#!/usr/bin/env python


import os

import time


max_mem = 0;

PID = raw_input("Please input your target PID:")

print '      The max_RAM used during this stage is:'

while True:

    fd = os.popen('ps aux')

    fd.readline()


    for line in fd:

        tmp_list = line.split()

        if tmp_list[1] == PID:

         if max_mem < float(tmp_list[3]):

                     max_mem = float(tmp_list[3])

                     print '        ',max_mem


    time.sleep(1)
```

Supplementary file 5. A perl script was implemented for computational simulation for *in silico* data

```
#!/usr/bin/perl -w


=pod

This Perl script generate simulated single-end short reads,

written by Yifei Tang,

from Center for Systems Biology, Soochow University,

with no warranty.

We used part of thought by Juliane Dohm and Claudio Lottaz.

=cut


$Usage = <<EOF;

Users can simply run this script followed by 5 arguments:

        1. Genome sequence file in Fasta format which you will generate reads from.

        2. Number of reads to be generated.

        3. Length of each read.

        4. Assumed sequencing error rate (%).

        5. Assumed sequencing indels rate (%).

        6. Indels length.

        7. Base name of output files. (<basename>_SE.fa)

Eg. perl simulation_SE.pl hg.fa 50000 36 0.8 0.0001 4 simread

This command will generates 50000 36-length short reads from "hg.fa

with sequencing error rate 0.8%, output to simread_SE.fa.

EOF


#Check if a leagal command line is given.

die "$Usage\n" if (@ARGV != 7);
```

```
#Define variables.

my $in = $ARGV[0];

my $totalreads = $ARGV[1];

my $readlen = $ARGV[2];

my $errorrate = $ARGV[3];

my $insertionrate = $ARGV[4];

my $deletionrate = $ARGV[4];

my $indellength = $ARGV[5];

my $basename = $ARGV[6];

my (@seq, @rev_seq, @chro, @chro_len, @chro_reads);

my $chro_index = 0, $total_len = 0, $errors = 0, $read_index = 0;


#Open input and output files.

open (IN, $in) || die "Genome sequence file not found!\n";

open (OUT, ">$basename\_SE.fa");

open (INDEL, ">indels.txt");


#Read the genome sequence file line by line.

print "Reading genome sequence...\n";

while (<IN>) {

    chomp;

    $_ =~ s/[\n\r]//;

    if ($_ =~ /\>/) {

        if ($chro_index != 0) {

            my $rev_seq = reverse ($seq[$chro_index]);

            $rev_seq =~ tr/ATCGU/TAGCA/;
```

```
            $rev_seq =~ tr/atcgu/tagca/;
            $rev_seq[$chro_index] = $rev_seq;
            $chro_len[$chro_index] = length ($seq[$chro_index]);
            $total_len += $chro_len[$chro_index];
            print "Length of chromsome $chro_index... \"$chro[$chro_index]\" is $chro_len[$chro_index]\n";
        }
        my $chro = substr ($_, 1, length ($_));
        $chro[++$chro_index] = $chro;
        print "Processing chromsome $chro_index... \"$chro\"\n";
    }elsif ($_ =~ /[ATCGUatcguNn]/) {
        $seq[$chro_index] .= $_;
    }
    if (eof) {
        my $rev_seq = reverse ($seq[$chro_index]);
        $rev_seq =~ tr/ATCGU/TAGCA/;
        $rev_seq =~ tr/atcgu/tagca/;
        $rev_seq[$chro_index] = $rev_seq;
        $chro_len[$chro_index] = length ($seq[$chro_index]);
        $total_len += $chro_len[$chro_index];
        print "Length of chromsome $chro_index... \"$chro[$chro_index]\" is $chro_len[$chro_index]\n";
    }
}
print "Complete with reading genome sequence with the total length of $total_len.\n";
#Determine reads to be generated of each chromsome.
print "Determining reads to be generated of each chromsome...\n";
for (my $n = 1; $n <= $chro_index; $n++) {
    $chro_reads[$n] = int (($chro_len[$n] / $total_len) * $totalreads);
```

```
	print "Chromsome $chro[$n] will generate $chro_reads[$n] reads.\n";
}
print "Complete with determing reads to be generated of each chromsome.\n";
```

```
#Main part simulating short reads.
print "Generating short read pairs...\n";
for (my $n = 1; $n <= $chro_index; $n++) {
	for (my $m = 0; $m < $chro_reads[$n]; $m++) {
		$read_index++;
		my $start = int (rand ($chro_len[$n] - $readlen));
		my ($ifrev, $errorIndex, $errorBase);
		my ($read, $errorread, $indelread);
		my ($adjustedOriSeq, $adjustedPostSeq);
		if (rand (1) >= 0.5) {
			$read = substr ($seq[$n], $start, $readlen);
```

```
            $ifrev = "+";
        }else {
            $read = substr ($rev_seq[$n], $start, $readlen);
            $ifrev = "-";
        }
        #Generating sequencing error
        ($errorread, $errorIndex, $errorBase) = &seq_error ($read);
        #Generating indels
        ($indelread, $adjustedOriSeq, $adjustedPostSeq) = &indels ($errorread);
        #Checking if uncertain bases reach the number of 10 (N)
        my $uncertainbases = 0;
        foreach my $base (split (//,$indelread)){
            $uncertainbases++ if ("\U$base" eq "N");
        }
        if ($uncertainbases >= 10) {$read_index--; redo;}
        if ($errorread ne $indelread) {
            print INDEL ">$read_index\_$chro[$n]\_$start\n$adjustedOriSeq\n$adjustedPostSeq\n";
        }
        if ($read ne $errorread) {
            ++$errors;
            chop ($errorIndex), chop ($errorBase);
            print OUT ">read_$read_index\_$chro[$n]\_$start\_$errorIndex\_$errorBase\n$indelread\n";
        }else {
            print OUT ">read_$read_index\_$chro[$n]\_$start\n$indelread\n";
        }
    }
print "Complete with generating short reads from $chro[$n].\n";
```

```
}

print "Complete with generating all short reads.\n";

print "You get $errors errors out of $read_index of all generated reads.\a\n";



#Close files opened.

close IN;

close OUT;

close INDEL;
```

```
#Subroutine processing sequencing error.

sub seq_error {

        my $tmpread = $_[0];

        my @base = split (//, $tmpread);

        $tmpread = "";

        my $errorIndex = "";

        my $errorBase = "";

        for (my $n = 0; $n < @base; $n++) {

                if (rand (100) > $errorrate) {

                        $tmpread .= $base[$n];

                }else {

                        my %fourbase = ("A", 0, "T", 0, "C", 0, "G", 0);

                        delete $fourbase{"\U$base[$n]"};

                        my @fourbase = keys (%fourbase);

                        my $index = $n + 1;

                        my $i = rand (1);
```

```
                if ($i < 0.33) {
                    $tmpread .= $fourbase[0];
                    $errorIndex .= "$index,";
                    $errorBase .= $base[$n] . "->" . $fourbase[0] . ",";
                }elsif ($i <0.66) {
                    $tmpread .= $fourbase[1];
                    $errorIndex .= "$index,";
                    $errorBase .= $base[$n] . "->" . $fourbase[1] . ",";
                }else {
                    $tmpread .= $fourbase[2];
                    $errorIndex .= "$index,";
                    $errorBase .= $base[$n] . "->" . $fourbase[2] . ",";
                }
            }
        }
    return ($tmpread, $errorIndex, $errorBase);
}


                #Deleting deletion sequence
                for ($n = 0; $n < $indellength; $n++) {
                    $adjustedOriSeq .= $base[$i+$n];
                    $adjustedPostSeq .= "-";
                }
                $i = $i + $indellength - 1;
            }else {
                $postSeq .= $base[$i];
                $adjustedOriSeq .= $base[$i];
```

```
                $adjustedPostSeq .= $base[$i];
            }
        }
        return ($postSeq, $adjustedOriSeq, $adjustedPostSeq);
}
```

```
#Subroutine processing indels.
sub indels {
        my $oriSeq = $_[0];
        my $postSeq = "";
        my $adjustedOriSeq = "";
        my $adjustedPostSeq = "";
        my @base = split (//, $oriSeq);
        my @fourbase = ("A", "T", "C", "G");
        for(my $i = 0; $i < @base; $i++) {
            if (rand (100) <= $insertionrate) {
                print "Insertion happens.\n";
                #Generating random insersion sequence
                for ($n = 0; $n < $indellength; $n++) {
                    my $randIndice = rand (1);
                    if ($randIndice < 0.25) {
                        $postSeq .= $fourbase[0];
                    }elsif ($randIndice < 0.5) {
                        $postSeq .= $fourbase[1];
                    }elsif ($randIndice < 0.75) {
                        $postSeq .= $fourbase[2];
                    }else {
```

```
                    $postSeq .= $fourbase[3];

                }

                $adjustedOriSeq .= " ";

                $adjustedPostSeq .= "+";

        }

}

if (rand (100) <= $deletionrate) {

        print "Deletion happens.\n";
```
